# Understanding the impact of bioactive coating materials for human mesenchymal stromal cells and implications for manufacturing

**DOI:** 10.1007/s10529-023-03369-9

**Published:** 2023-05-25

**Authors:** Pedro Silva Couto, Samuel A. Molina, Denis O’Sullivan, Liam O’Neill, Alexander M. Lyness, Qasim A. Rafiq

**Affiliations:** 1 Department of Biochemical Engineering, Advanced Centre for Biochemical Engineering, University College London, Gower Street, London, WC1E 6BT UK; 2 Applied Research & Technology Scouting R&D, West Pharmaceutical Services, Inc., Exton, PA USA; 3 TheraDep, Questum, Ballingarrane, Clonmel, Co., Tipperary, Ireland

**Keywords:** Mesenchymal, Manufacturing, Biomaterials, Cyclic olefin polymer, Surface

## Abstract

Bioactive materials interact with cells and modulate their characteristics which enable the generation of cell-based products with desired specifications. However, their evaluation and impact are often overlooked when establishing a cell therapy manufacturing process. In this study, we investigated the role of different surfaces for tissue culture including, untreated polystyrene surface, uncoated Cyclic Olefin Polymer (COP) and COP coated with collagen and recombinant fibronectin. It was observed that human mesenchymal stromal cells (hMSCs) expanded on COP-coated plates with different bioactive materials resulted in improved cell growth kinetics compared to traditional polystyrene plates and non-coated COP plates. The doubling time obtained was 2.78 and 3.02 days for hMSC seeded in COP plates coated with collagen type I and recombinant fibronectin respectively, and 4.64 days for cells plated in standard polystyrene treated plates. Metabolite analysis reinforced the findings of the growth kinetic studies, specifically that cells cultured on COP plates coated with collagen I and fibronectin exhibited improved growth as evidenced by a higher lactate production rate (9.38 × 10^5^ and 9.67 × 10^5^ pmol/cell/day, respectively) compared to cells from the polystyrene group (5.86 × 10^5^ pmol/cell/day). This study demonstrated that COP is an effective alternative to polystyrene-treated plates when coated with bioactive materials such as collagen and fibronectin, however COP-treated plates without additional coatings were found not to be sufficient to support cell growth. These findings demonstrate the key role biomaterials play in the cell manufacturing process and the importance of optimising this selection.

## Introduction

Cell and gene therapy (CGT) is a developing field of medicine that focuses on whole cells or their secreted products (such as extracellular vesicles) as active ingredients. Multiple cellular candidates such as T-cells, NK-cells, hematopoietic stem cells (HSCs) or human mesenchymal stromal cells (hMSCs) have populated the clinical trial landscape in the last decade (Culme-Seymour et al. 2012; Heathman et al. 2015; Silva Couto et al. 2017; Couto et al. 2019; Kabat et al. 2020). Due to their immunomodulatory properties and in vitro differentiation ability, hMSCs’ safety and efficacy have been studied in several clinical trials targeting a plethora of conditions from graft-*versus* host disease (GvHD) (Li et al. 2022) to acute myocardial infarction (AMI) (Attar et al. 2021) and Crohn’s Diseases (Vieujean et al. 2022) amongst others.

Process development and manufacturing optimisation play a key role in translating small-scale lab-based therapeutics to large-scale commercialisation. From an engineering perspective, the challenge is to ensure that all the necessary bioprocessing steps performed during the manufacturing process are scalable. Given that hMSCs are anchorage-dependent cells, surfaces or matrixes need to be provided to ensure cell proliferation. While it has been possible to expand hMSCs as monolayers on simple polystyrene surfaces, the preferred approach is to provide a surface which has been altered to maximise cell proliferation. This has given rise to a range of surface treatments and coatings that include protein layers such as collagen, fibronectin, vitronectin, laminin and mixtures of biomolecules. Previous research demonstrated that collagen was responsible for promoting hMSC adhesion and proliferation when compared to polystyrene coated plates (Somaiah et al. 2015; Salzig et al. 2016). Other studies have also evaluated the impact of molecules such as fibronectin, which was previously reported to enhance cell attachment and modulate cell differentiation (Veevers-Lowe et al. 2011; Somaiah et al. 2015; Salzig et al. 2016; Hsiao et al. 2017). While fibronectin is involved in cell adhesion and cell migration processes, collagen has a critical role to ensure a regulated functioning of the connective tissues throughout the body. Providing this base layer of protein allows cells to accelerate cell adhesion, spreading and growth on the surface (Somaiah et al. 2015; Lerman et al. 2018). Due to their simplicity of use, monolayer culture has been the backbone of the initial studies in the CGT industry. This is a strategy was used to conduct preliminary or small-scale studies.

To address the unmet need for scalability, typically suspension-based bioreactor processes are preferred. However, due to the adherent nature of hMSCs, microcarriers are often required for the cells to grow in suspension. This approach involves supplementing the culture with microcarrier particles or beads which can be used as surfaces for the cells to adhere to. There is a multitude of microcarriers which are commercially available and provide a range of different diameters, coatings, densities and surface charge. Therefore, it is important to evaluate which microcarrier formulation is optimal for a specific cell type (Rafiq et al. 2016). Adherent cell types often require basolateral signals to simulate a healthy environment in which to thrive. Many of these signals come from proteins found in the extracellular matrix. Of these matrix proteins, fibronectin, laminin, various types of collagens, and proteoglycans are often used to stimulate adherent cell attachment and subsequent outgrowth. With regards to the microcarrier coatings, although a large number of studies have used polystyrene-based microcarriers (Rafiq et al. 2013, 2017a, b; Dos Santos et al. 2014; Petry et al. 2016; De Soure et al. 2017; Silva Couto et al. 2020), there are several studies using collagen-coated microcarriers (Sun et al. 2010; Yuan et al. 2014; Mizukami et al. 2016). Others have chosen microcarriers manufactured with dextran (Schop et al. 2010; Chen et al. 2015; Shekaran et al. 2015; Lam et al. 2017) or microcarriers coated with recombinant fibronectin (Schirmaier et al. 2014). The multiplicity of microcarrier coatings used in these studies suggests a lack of consensus regarding which coating enhances cell growth and cell potency, however, oftentimes the type of cell being cultured must be expanded on extracellular matrices that mimic the natural physiologic environment to retain functionality.

Cyclic olefin polymer (COP) as a material, possesses inert qualities that make it suitable for contact with living drugs, such as low extractables profile and flexibility of manufacturing (Niles and Coassin 2008; Waxman et al. 2017; Floyd et al. 2020). Whilst the inert nature of COP provides exceptional stability for cell culture studies, it provides challenges for surface functionalisation (Niles and Coassin 2008). COP has been successfully surface-modified previously using coronal-discharge or plasma-enhanced chemical vapor deposition of to form surface coating of silicone oxide (Dudek et al. 2009). Such plasma approaches are required to overcome the inherent stability of the base plastic. While new modifications are being actively pursued to enable new uses (Shim et al. 2019), the majority of surface modification technologies for COP still rely upon plasma based activation to produce adequate coating adhesion. While the use of COP for medical devices and pharmaceutical containers is widely accepted for small molecules and biologics, its biocompatibility with cells using established testing guidelines is not fully known. Microneedles made of COP were tested for off-target effects on human primary skin cells but were found to be non-toxic to both keratinocytes and endothelial cells (Schossleitner et al. 2019).

It is also possible to coat COP with biologically active coating materials. Coatings are useful for a number of reasons. Anti-fouling is a desirable trait for any device that in implanted or left in place for longer periods of time, like catheters, stents, and pacemakers. Lubricious coatings provide the necessary movement for devices with tight dimensions, like syringes used in pumps and lubricant-free valves. Biologically active coatings present a stability and functional challenge since many of these bioactive materials, such as proteins, are directly affected by heat, pH, and the effects of electricity (Olatunde et al. 2021).

From a bioprocessing perspective, the investigation of different bioactive coatings is important to support both monolayer and microcarrier culture of hMSCs. Although the vast majority of studies using either culture strategies employ culture-treated polystyrene surfaces (Bruder et al. 1997; Banfi et al. 2000; Bonab et al. 2006; Bertolo et al. 2016), there is increasing evidence emerging about the potential role of different bioactive coatings such as collagen or recombinant fibronectin (Singh and Schwarzbauer 2012; Maerz et al. 2016; Basoli et al. 2021) to enhance cell proliferation (Somaiah et al. 2015; Salzig et al. 2016; Smeriglio et al. 2017).

Traditionally, the bioactive coating process usually begins with a plasma treatment, to enhance hydrophilicity, followed by multi-step aseptic techniques to deposit thin bioactive layers in sterile processing conditions. Despite the complexity and challenges this presents, these methods have become well established and represent the standard surfaces for growing hMSCs on polystyrene surfaces. However, these methods often struggle to provide adequate coatings on less reactive plastics such as COP (O’Sullivan et al. 2020c).

One potential route to functionalising COP substrates is the use of cold plasma deposition. Recent studies have shown that a plasma—aerosol process can effectively deposit and adhere collagen coatings onto cell culture surfaces with enhanced cellular responses (O’Sullivan et al. 2020c).

In this study, we assess the compatibility of hMSCs grown on COP coated with biologically active proteins using a novel coating cold-plasma deposition technique. This approach combines the plasma activation of the COP surface with the ability to adhere functional coatings to the surface in a single process. The study aims to compare the growth and metabolic profile of hMSCs on different COP treated plates including: (i) COP treated with cold-deposition plasma (TA), (ii) COP coated with collagen type I (TB), (iii) COP coated with fibronectin (TC), and (iv) COP coated with anti-CD3 (TD). These were compared with traditional plasma gas tissue culture treated polystyrene (PS) plates and COP non-treated plates (Untreated). In the present study, it was hypothesised that the presence of bioactive coatings would improve hMSC growth kinetics and metabolism, with the collagen and fibronectin coatings, in particular, possibly promoting greater attachment between cells and the matrices, potentially leading to enhanced growth kinetics.

## Materials and methods

### Plates and coatings

The coating terminology used throughout is summarised in the following paragraph. Polystyrene tissue culture treated plates were labelled as “PS” (commonly used in adherent cell culture). Instead of polystyrene, the plates referred to as “Untreated”, “TA”, “TB”, “TC” and “TD” were manufactured with Crystal Zenith® (CZ) COP. These plates were treated with cold-deposition plasma (TA), coated with collagen type I (TB), fibronectin (TC), or anti-CD3 (TD), using a cold plasma deposition method described in detail elsewhere (O’Sullivan et al. 2020a, b, c). In summary, plasma deposition was carried out using a Biodep unit manufactured by TheraDep Inc. This is a purpose-built deposition system comprising a Redline G2000 High Voltage (HV) generator connected a custom machined Teflon block that encased two metal electrodes. For these experiments, the HV generator was operated at an output voltage of 120 V and 20 kHz and with a 45% duty cycle. A helium flow of 6 slm was provided to the metal electrodes to create the plasma discharge. To deposit a coating, a pneumatic nebulizer (T2100 from Burgener Research, Canada) was placed between the electrodes, and this was connected to a syringe pump to provide a constant flow of biomolecule solution at 40 µl/min. The liquid was nebulized using a gas flow of approximately 2 slm. The plasma discharge was combined with the nebulized droplet spray in an acrylic tube (19 mm inner diameter × 35 mm length) and the substrates to be coated were placed at the outlet of the tube. To produce different coatings, the biomolecule solution was changed. Collagen (3 mg/ml, bovine Type I, from Collagen Solutions, UK), fibronectin from human plasma (Sigma Aldrich, 3 mg/ml in sterile water) and mouse anti-human CD3 Clone UCHT-1 (BD Biosciences,1 mg/ml in tris buffered saline) were introduced directly into a non-thermal plasma discharge and deposited directly onto the COP plates using an automated plasma spray coating system. This deposited approximately 2 µg/cm^2^ for the collagen and fibronectin coatings, but only one approximately 0.7 µg/cm^2^ on the anti-CD3 surfaces. The Untreated control group refers to bare COP plates with no additional treatment or coating performed.

### Cells and medium formulations

To perform this work, the hMSCs originated from a male healthy donor of 22 years old of unknown ethnic background were used. A vial of hMSCs (Roosterbio, US) was expanded in monolayer for two passages and cryopreserved at the density of 1 × 10^6^/ml using CryoStor® CS10 (hereafter referred to as CS10) complete freezing medium (BioLife Solutions, US). After CS10 addition, the vials were placed into Nalgene® Mr Frosty (Merck, UK) storage containers and stored at − 80 °C overnight. On the following day, the vials were transferred to liquid nitrogen. The cells used in this work were stored at 10.31 population doubling levels (PDLs) which corresponded to P2.

**Fig. 1:**
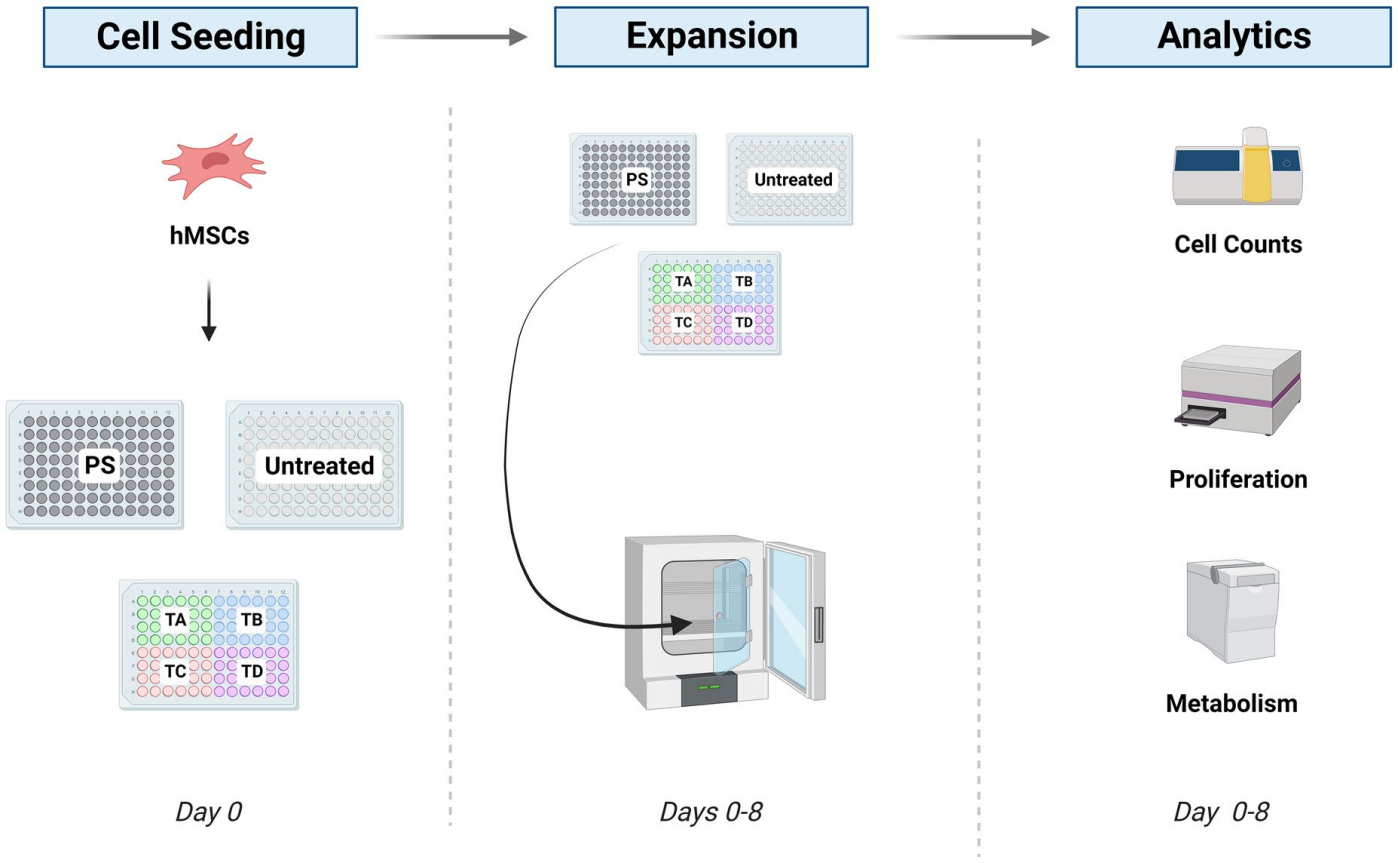
Schematic representation of the different stages of the study aiming to evaluate the impact of multiple coatings on growth kinetics and metabolic profile of hMSCs. TA refers to COP plates coated with cold-deposition plasma, TB refers to COP plates coated with collagen type I, TC refers to COP plates coated with fibronectin (TC), and TD refers to COP plates coated with anti-CD3. These were compared with traditional plasma gas tissue culture treated polystyrene (PS) plates and non-tissue culture treated (Untreated) COP plates

Cells were cultured using Dulbecco’s Modified Eagles Medium (DMEM, 1 g/l glucose; Lonza, UK) supplemented with 10% (v/v) foetal bovine serum (FBS) (Gibco, US) and 1 mM UltraGlutamine (Lonza, UK) (hereafter referred to as expansion medium). The cells were seeded at a density of 5000 cell/cm^2^ with no medium exchange performed during the experiment. During the eight days of culture, the cells were kept at 37 °C and 5% CO_2_ using a humidified incubator (PHCbi, Singapore). At the end of the expansion period, several analytics were performed as described in the section below. The design of this study as well as the types of plates used are summarised in Fig. 1.

### Analytical techniques

Phase-contrast imaging was used to assess cell morphology across different plates. An EVOS phase-contrast microscope (ThermoFisher, UK) was used to perform daily imagining at × 4, × 10, × 20 and occasionally × 40 magnification.

Cell counting and viability determination were performed daily using a NucleoCounter NC-3000 and Via1-Cassette™ (Chemometec, Denmark). These cassettes have acridine orange (AO) and 4′6-diamidino-2-phenylindole (hereafter referred to as DAPI) immobilised to detect cells and non-viable cells. AO is a permeable dye that binds directly to cell nuclei, whereas DAPI can only enter damaged cell membranes.

As hMSCs are an adherent cell type, for every cell count, the following protocol was performed. Cell culture supernatants were removed and stored for metabolite analysis. After a washing step was performed using PBS, the cells were detached with 0.25% (v/v) trypsin and 0.02% (w/v) EDTA solution (ThermoFisher, UK). A 5-min incubation period was performed (37 °C, and 5% CO_2_). Using a phase-contrast microscope, it was possible to confirm whether the majority of the cells were detached from the surface of the well plates. Expansion medium was added to each well (on 1:2 ratio to trypsin volume) with the total content then transferred to a centrifuge tube for pellet concentration. The centrifugation cycle parameters were 400xg and 5 min (Eppendorf, US). Once pelleted, the supernatant was removed, a fresh expansion medium was added, and the cell count was performed.

One of the critical aspects of this study was assessing how each coating impacts cell proliferation. Additionally, an indirect cell concentration determination method approach was included, as the cell counting protocol consists of a supernatant removal step, which may contain floating cells that are still viable. This assay enables the comparison of indirect live cell concentrations, regardless of whether they are adherent or suspended. Due to the small-scale nature used to conduct this study, relying exclusively on the NC-3000 data may not be sufficient as multiple reads could fall outside the equipment's optimal reading range for cell concentration (5 × 10^4^ to 5 × 10^6^ cells/ml). To overcome the limitations of automated cell counts when working at low concentrations, the WST-1 assay (Roche, Switzerland) was performed daily. This colorimetric test uses the reduction of WST-1 by viable cells as an indirect measure for viable cell concentration determination. WST-1 assay features a stable tetrazolium salt that is cleaved leading to a soluble form of formazan in a process occurring at the cell surface level. The amount of formazan dye in the supernatant can then be correlated to the metabolically active number of cells. Briefly, the WST-1 was added to the tissue culture well plates in a volumetric ratio of 1:10, without a previous washing step being performed, according to the manufacturer’s protocol. The sample was kept for one hour in a humidified incubator at 37 °C and 5% CO_2_. The samples were then analysed using a plate reader Infinite® 200 PRO (Tecan, Switzerland). Due to the need of performing cell detachment steps to measure the viable cell concentration, the indirect cell evaluation steps were performed using additional plates.

Glucose metabolism is known to be linked to cellular stress responses (Zhao et al. 2008), so glucose concentration was monitored as an indicator of cellular stress response to the various treatments. To analyse glucose and lactate concentration, the cell culture supernatants were aspirated before cell detachment and stored in a freezer with the temperature set to − 80 °C. The triplicates from the same sample were pooled to meet the minimum volume required to run the assay. Then the protocol for glucose and lactate determination was performed using the automated platform CubiAn Bioanalyzer (Optocell GmbH, Germany).

### Equations

Different equations were used to compare the growth kinetics and metabolism of the cells seeded in the different plates/coatings used in this study.

1 $$\mu =\frac{Ln\left(\frac{cx\left(t\right)}{cx\left(0\right)}\right)}{\Delta t}$$

where μ represents the specific growth rate (d^−1^), Cx(t), and Cx(0) describe the total cell numbers at the end and the start of the exponential growth phase, respectively. Time was represented by t (d).

2 $${t}_{d}=\frac{Ln (2)}{\mu }$$

where t_d_ represents doubling time (d) and μ represents the specific growth rate (d^−1^)

3 $${q}_{met}=\frac{\mu }{cx\left(0\right)}\times \frac{{C}_{met\left(t\right)}-{C}_{met\left(0\right)}}{{e}^{\mu t}-1}$$

where q_met_ represents specific metabolic consumption/production rate, µ specific growth rate (d^−1^), C_met(0)_ and C_met(t)_ correspond to the metabolite concentration at the start and end of the exponential growth phase, respectively. Cx(0) is the cell number at the beginning of the exponential growth phase, whereas t represents time.

### Statistical analysis

Unless explicitly stated otherwise the data herein presented was shown as mean with error bars representing standard deviation from triplicate measurements (i.e. N = 3). Unless otherwise stated, statistical analysis between groups was performed using a non-parametric test (Kruskal–Wallis) following by a post hoc analysis based on Dunn’s post hoc test. The statistical analysis was performed using GraphPrism Version 8.3.0 (Graphpad Software, US). Differences were considered statistically significant with a P-value below 0.05. Significance levels were set at P-values < 0.05 (*P-value < 0.05, **P-value < 0.0.01, ***P-value < 0.001).

## Results

### Cell counts, viability, and proliferation assessment

To determine the impact of different bioactive coatings on the growth and metabolism of hMSCs, the cells were expanded in COP 96-multiwell plates treated with the six different surface modifications: TA, TB, TC, TD, traditional culture-treated polystyrene (PS) and untreated (Untreated) bare COP plates. As previously described, the cells were seeded at a density of 5000 cell/cm^2^. The battery of analytics used to assess, growth kinetics and metabolic profile included, cell count and viability measurements, indirect proliferation assay (WST-1) and glucose/lactate concentration assay. Viable cell concentration and viability measurements were performed to evaluate how each plate or coating interacted with the hMSCs. Additionally, an indirect form of cell proliferation was performed (WST-1) to validate the trends from the offline cell counts and to evaluate the presence of viable non-adherent cells.

**Fig. 2:**
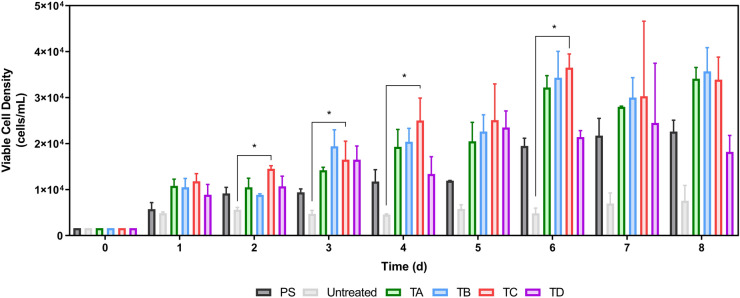
Viable cell density during the 8-day expansion period using BM-hMSC plated over polystyrene (PS), untreated and plates coated with TA, TB, TC and TD. Data shown as mean with error bars representing standard deviation (N = 3)

Figure 2 provides the viable cell concentration observed across all plates over an 8-day culture period. With respect to cell concentration, it was observed that all coatings employed in this study, with the exception of the untreated plates, resulted in some level of cell growth during the culture, with some coatings resulting in significantly higher levels (P-value < 0.05) of growth compared to others (Fig. 2). As expected, while the positive control (PS) demonstrated cell growth over the culture period resulting in approximately ~ 2.2 × 10^4^ cells/ml, the same was not true for the negative control (Untreated) which resulted in approximately ~ 0.5 × 10^4^ cells/ml, only a slight increase above the initial cell seeding density (5000 cell/cm^2^). However, hMSC cultured on coatings TA (COP treated with cold deposition plasma), TB (COP collagen-coated plates) and TC (COP fibronectin-coated plates) resulted in the highest cell densities by the end of the culture with cell densities of approximately ~ 3.3, 3.5 and 3.2 × 10^4^ cells/ml respectively. The same was not true for TD (COP treated with anti-CD3), which achieved comparable cell concentrations to the positive control (PS) of approximately ~ 2 × 10^4^ cells/ml. The data also suggests that the maximum cell concentration was achieved between days 6 and 8 days of culture regardless of the coating or plate used. The cell growth kinetics, specifically the growth rate and doubling time (Table 1) reflect the difference between coatings. It was observed that the Untreated group led to significantly higher doubling time than PS, TD (P-value < 0.05) and TA, TB, TC (P-value < 0.01).

**Table 1:** Summary of the growth kinetics parameter calculations for cells seeded in different plates or coatings

Group	Growth rate, d^−1^ (Mean ± STDEV)	Doubling time, d (Mean ± STDEV)	P-value
PS	0.151 ± 0.021	4.64 ± 0.60	< 0.05
Untreated	0.087 ± 0.010	8.07 ± 0.97	–
TA	0.193 ± 0.018	3.64 ± 0.34	< 0.01
TB	0.231 ± 0.023	3.02 ± 0.31	< 0.01
TC	0.297 ± 0.028	2.78 ± 0.30	< 0.01
TD	0.177 ± 0.054	4.13 ± 0.63	< 0.05

P-values reported between Untreated group and all the other conditions tested

**Fig. 3:**
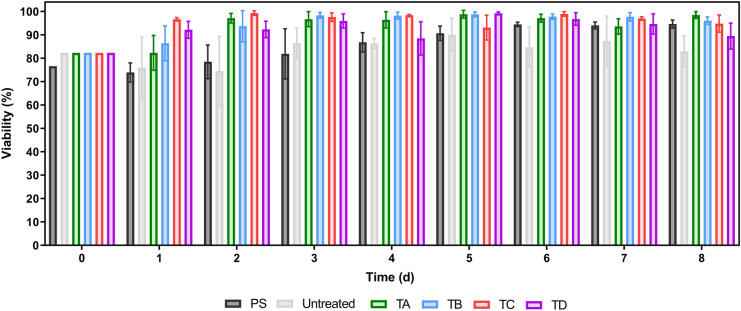
Viability during the 8-day expansion period using BM-hMSC plated over polystyrene (PS), untreated and plates coated with TA, TB, TC and TD. Data shown as mean with error bars representing standard deviation (N = 3)

Viability determination was performed alongside the cell growth kinetics (Fig. 3). Given the adherent nature of hMSCs, a cell detachment steps are required prior a cell count was performed. Due to the way it was designed, the WST-1 assay enables the quantification of quantifying the signal (in the form of absorbance) from cells that could be in suspension and eventually, still metabolically active. It was observed that every plate/coating included in the study resulted in > 90% viability by day 8 with the exception of the Untreated condition. It is noteworthy that in addition to generating the highest cell densities, the coatings TA, TB and TC resulted in the highest levels of cell viability by the end of the culture, including the culture-treated PS control condition. This increase in viability was noticeable after day 1 for TC and day 2 for TA and TB. It should be highlighted that after eight days, cells cultured with TA, TB and TC were more than 95% viable, whereas this was not observed either for PS (below 95%) neither in the Untreated and TD groups (below 90%). An analysis of the viability change over time showed that it takes a minimum of 5 days for PS-cultured hMSC to reach 90% viability. In contrast, similar viability levels were reported at the 2-day time point for TC and TD plates and after three days for groups TA and TB (Fig. 3).

**Fig. 4:**
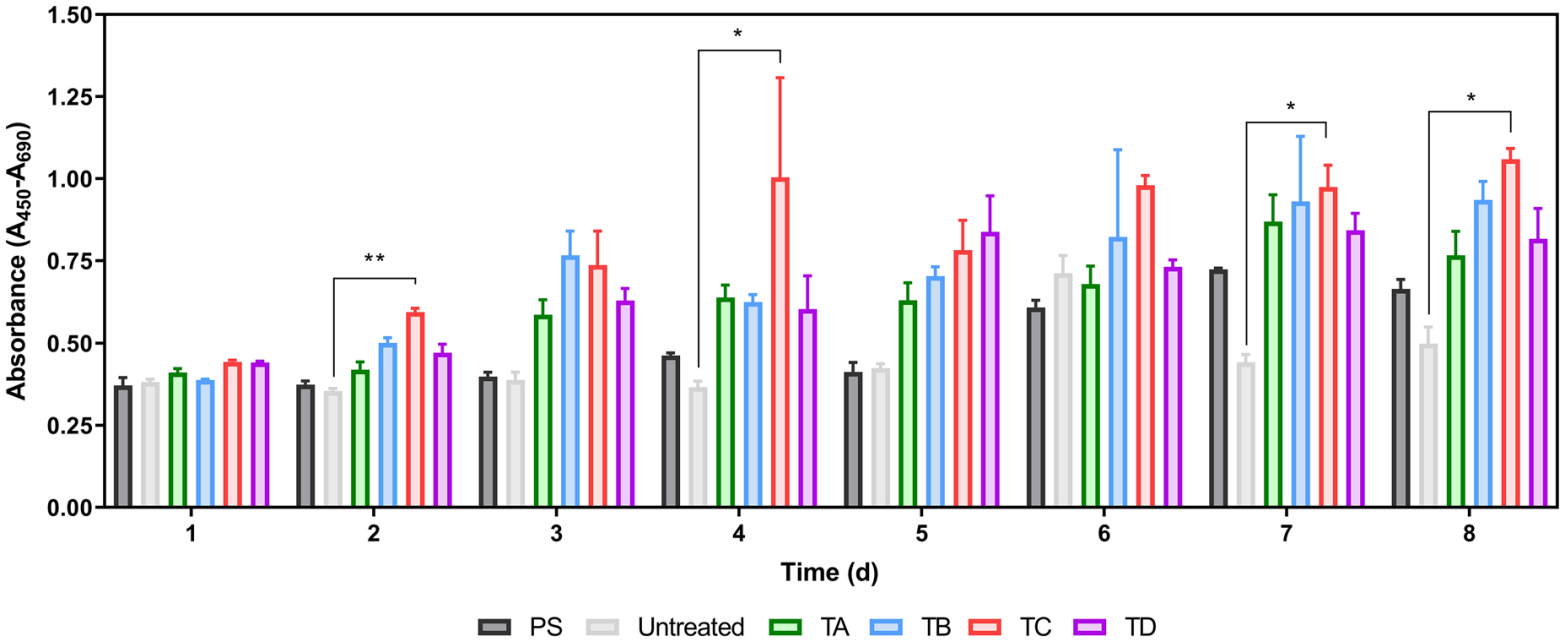
Indirect viable cell quantification during the 8-day expansion period using BM-hMSCs plated over polystyrene (PS), untreated, and plates coated with TA, TB, TC and TD. Data shown as mean with error bars representing standard deviation (N = 3). To perform an indirect estimation of the viable cell concentration the WST-1 assay featuring a tetrazolium salt (4-[3-(4-Iodophenyl)-2-(4-nitro-phenyl)-2H-5-tetrazolio]-1,3-benzene sulfonate) was performed

To validate our offline cell counts and evaluate the presence of viable cell populations potentially in the suspension fraction, the WST-1 assay was performed (Fig. 4). The WST-1 and cell counting assays focus on different biological principles that are complementary. Whereas the NC-3000 cell count and viability approach uses a two-dye system to perform the cell counts (DAPI and acridine orange), the WST-1 converts a salt dissolved in the medium. The results from the WST-1 assay (Fig. 4) reinforce the trends obtained in the cell count and viability data (Figs. 2 and 3) with the absorbance values increasing over time, and more specifically, that coatings TA, TB and TC reached higher absorbance values from day two onwards, indicating greater cell proliferation when compared with PS, Untreated and TD. Based on this analytical technique, PS and TD groups result in similar cell growth levels, whilst the Untreated plate led, once again, to the poorest growth in the study.

#### Morphology analysis

In addition to cell counts and proliferation assessment, phase-contrast microscopy was performed to assess the presence of phenotype changes introduced by any of the coatings/plates included in the study (images not shown). It was possible to observe that the cells plated in polystyrene plates (positive control) exhibited the spindle shape that characterises hMSC (Dominici et al. 2006). As expected, this morphology was not observed when the cells were seeded in Untreated plates. Due to the inability to attach to the surface of the cell culture plate, it appears that cells tend to aggregate in clusters. It was also observed that the morphological analysis matches the cell counting and proliferation assay information where coatings TA, TB and TC appear to lead to higher confluency whereas Untreated appear to lead to substantially lower confluency. An exception should be made for TD coated plates. Although cells exhibited the spindle shape that characterises hMSC (Dominici et al. 2006), cell density seems relatively lower than in TA, TB and TC but comparable to PS at a similar time point.

#### Metabolic profile evaluation

**Fig. 5:**
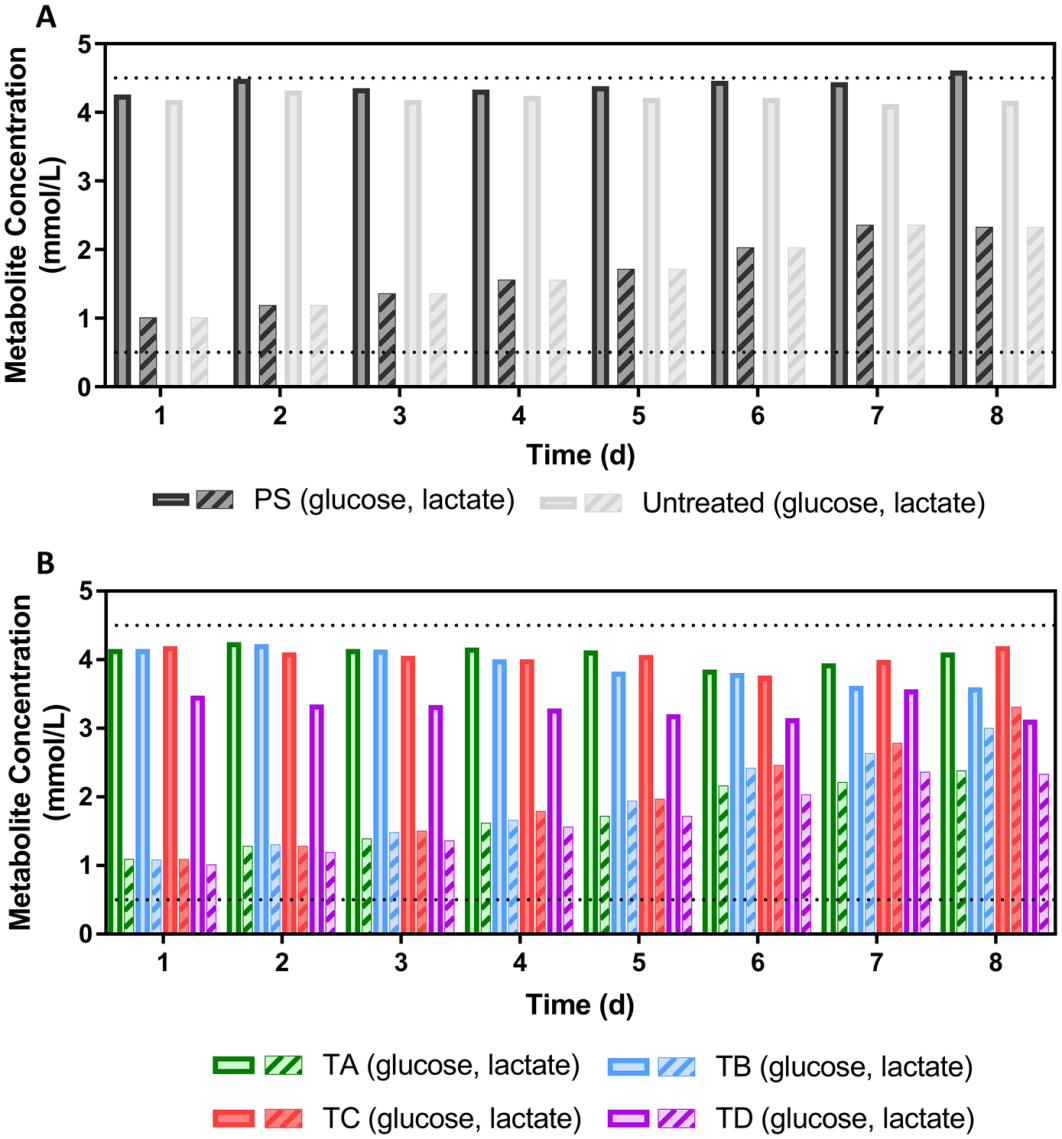
Cell supernatant metabolite quantification during the 8-day expansion period using BM-hMSCs plated over polystyrene (PS), Untreated, and plates coated with TA, TB, TC and TD. Data shown as mean with error bars representing standard deviation (N = 3). Horizontal dotted lines represent the read values for glucose (top) and lactate (bottom) at seeding time

To study the metabolic profile of the hMSCs when seeded on different coatings, glucose and lactate concentration were measured in the cell culture supernatants resulting from each plate used in the study (Fig. 5). It was observed that regardless of the plate or coating used, the lactate concentration found in the supernatant of each group increased when compared to the baseline. The lactate build-up suggests that cells are metabolically active in all the groups present in the study. The same does not seem to be valid in the Untreated group, where lactate may be produced to ensure cell survival. Previous research has demonstrated that that amino acids consumed at low rates account for most of carbon in cells (Hosios et al. 2016) which is aligned with the hypothesis that, in the context of this study, glucose may be used for to maintain cell survival.

Additionally, it was noticed that although the lactate concentration in the supernatants collected from TA and TD was comparable to the control groups (PS, Untreated), a higher concentration was reported in the supernatants from TB and TC. This trend aligns with the cell counting and proliferation data that seem to favour coatings TB and TC.

The glucose concentration did not show a sharp decrease after the 8-day expansion compared to the baseline. This observation may be due to two main reasons: (1) limitations of the small-scale nature of this study, (2) stoichiometric relationship between glucose and lactate production in glycolysis (Vazquez et al. 2010; Melkonian and Schury 2021). The small-scale nature of this study presented several challenges related to the analytical techniques available to be used. In this study, the highest glucose consumption rate was reported in the group TD. Also, it was possible to observe that although comparable from a growth kinetics perspective, hMSCs seeded in TB consume more glucose per cell and unit of time than cells from TA and TC groups (Table 2). This suggests that although growth kinetics may be similar, the metabolic pathways followed by cells when plated in different coatings may differ. The average glucose consumption rate from the hMSC expanded in PS plates was lower when compared to TD, group with a similar growth kinetic profile. The lactate production rate trend mimics the observations from the growth kinetic data with TA, TB, and TC with the highest reported rates.

**Table 2:** Summary of the average metabolite consumption/production rates from hMSCs seeded in different plates or coatings

Group	Glucose consumption rate, pmol/cell/day (Mean ± STDEV)	P-value	Lactate production rate, pmol/cell/day (Mean ± STDEV)	P-value
PS	(0.654 ± 0.47) × 10^5^	NS	(5.86 ± 1.70) × 10^5^	NS
Untreated	(1.77 ± 0.35) × 10^5^	–	(5.12 ± 0.91) × 10^5^	–
TA	(2.48 ± 0.75) × 10^5^	NS	(8.01 ± 2.21) × 10^5^	NS
TB	(3.41 ± 1.37) × 10^5^	NS	(9.38 ± 3.29) × 10^5^	NS
TC	(2.62 ± 0.74) × 10^5^	PNS	(9.67 ± 3.63) × 10^5^	NS
TD	(7.00 ± 0.88) × 10^5^	< 0.01	(7.96 ± 2.36) × 10^5^	NS

P-values reported between Untreated group and all the other conditions tested

## Discussion

To meet the high cell quantities required for CGT clinical applications, one of the critical process steps is the expansion phase, responsible for increasing the cell numbers during the manufacturing process. The increasing need for high cell quantities has led to the implementation of scalability studies (Rafiq et al. 2013; Schirmaier et al. 2014; Lawson et al. 2017). From a research perspective, this need has been addressed by conducting small-scale studies focused on cell growth kinetics optimisation modulating process parameters such as seeding density, medium formulation, protein coating and supplementation, albeit performed with standard tissue culture treated PS plastics (Nekanti et al. 2010; Wu et al. 2015; Oikonomopoulos et al. 2015; Bhat et al. 2021). While this data is well studied for PS culture flasks, our approach was to study whether different coating surfaces on a COP substrate have an impact on hMSC growth kinetics and their metabolism.

Similar experiments conducted in other research groups have shown that hMSCs’ doubling time can vary between 2 and 4 days (Schop et al. 2009; Chen et al. 2015; Shekaran et al. 2015; Petry et al. 2016). As previously reviewed, these parameters are modulated by multiple factors such as tissue source, medium formulation, seeding density, passage number, and other processed conditions (Silva Couto et al. 2020). While controlling for these factors, the results presented herein suggest that the plasma deposited coatings may have altered the growth rate of the cells. These results suggest that COP plates coated with TB and TC interact with the hMSC, increasing their growth potential. Previous reports have highlighted that collagen supports cell adhesion to surfaces. More recently, it was published that collagen promotes the adhesion and proliferation of hMSCs (Schor and Court 1979; Heino 2007). Additionally, the authors have reported that collagen substrates may be responsible for enhancing hMSCs’ osteogenic ability, which may benefit bone-related clinical applications (Somaiah et al. 2015). Similarly, fibronectin has been demonstrated to play a major role in cell adhesion and migration (Ruoslahti 1984; Ramos et al. 2016; Hsiao et al. 2017). Previous studies have reported that hMSC migration is also regulated by fibronectin (Veevers-Lowe et al. 2011). It can be hypothesised that the growth kinetic differences observed in this study may be due to the improvement of hMSC adhesion promoted by collagen and fibronectin (used to coat the plates of groups TB and TC, respectively). It should be mentioned that the CD3 was included in the study given its potential role in CD3 T cell expansion profile but not for adherent cell manufacturing. As such, the rational for including an anti-CD3 coating was to evaluate whether COP treated plates with an additional molecule with unknown properties could benefit adherent cell growth kinetics. The data presented suggests that the plasma process was able to deposit adherent protein layers directly onto inert COP surfaces without loss of biological functionality. Taken together these results highlight the opportunity of using plasma deposited collagen and fibronectin surfaces across different cell culture platforms such as cell culture plates, microcarriers, hollow fibre bioreactor cartridges and other scaffolds used for cell expansion and could enable a wide range of COP based cell growth surfaces. This current study highlights the opportunity that cold plasma-treated COP offers for the advanced therapy process development space, where utilising pharmaceutical grade single-use systems may be advantageous to cell-based products that often and currently see a multitude of plastics during manufacturing. One potential advantage to manufacturing could be the use of a single type of plastic whereby single-use systems may be impacting productivity in currently unknown ways. Cell culture treated COP single-use systems may solve this issue from start to end with well-known fill/finish aspects of pharmaceutical packaging options. It should be highlighted that the current technology enables the improvement of hMSC growth kinetics. However, the complex nature of the manufacturing and coating of COP treated plates was not developed to be performed by individual labs independently but in facilities with the knowledge and the equipment required to maximise the usage of this technology.

It should be highlighted the condition that Untreated plates lead to the lowest cell concentrations as well as lowest consumption and production rates of glucose and lactate respectively. It was hypothesised that these observations were mostly due to the inability of cells to adhere to the surface of the cell culture plate. Under these circumstances, cells tend to aggregate to avoid cell death due to the inability to attach to surfaces described as anoikis (Gilmore 2005). Anoikis has been previously reported in studies focused on spheroid usage as 3D models for tumour formation (Eguchi et al. 2015; Powan et al. 2017).

It is essential to  mention that the total cell concentrations used in this study are significantly smaller than when compared to monolayer studies conducted elsewhere (Schop et al. 2009; Chen et al. 2015; Shekaran et al. 2015; Petry et al. 2016) mainly due to the microplate study design. It is then pertinent to question whether glucose concentration differences are detectable at this low cell concentration. Ideally, an assay with higher sensitivity should be chosen. It is also important to mention that lactate concentration changes are generally more noticeable than glucose due to stoichiometric reasons. This relates to the fact that one mole of glucose in glycolysis is metabolised into two moles of lactate (Vazquez et al. 2010; Melkonian and Schury 2021). Based on cell counting and lactate concentration, it could be hypothesised that cells seeded in PS, TA, TB, TC are likely to be converting glucose into lactate for biomass production (i.e., cell growth). In an opposite direction, TD and Untreated conditions have led to comparable growth kinetics. However, given that the different metabolic rates between these two groups suggests that, in the TD group, glucose is being used for cell maintenance or survival rather than biomass production which suggests a mis-match of substrate and cell type.

Additionally, both control groups produced less lactate per cell and unit of time than the other groups tested. This result strengthens the case for plates coated with TA, TB and TC as they seem to optimise growth kinetics across different analytical techniques. Again, the high average lactate production rate of cells plated in TD compared to PS should be noted, despite comparable growth kinetics. This aligns with the hypothesis that hMSCs on TD plates consume glucose and produce lactate to ensure cell survival rather than biomass conversion.

## Conclusion

The CGT industry relies on the usage of different cell types, some of them adherent. This creates several manufacturing challenges, with scalability being one of them. Large scale expansion of hMSC typically relies on the usage of microcarriers. Different materials and coatings used in microcarrier manufacturing will inevitably lead to different growth kinetics and metabolic profiles. In this study, we evaluated COP plates coated with various bioactive materials in addition to traditional polystyrene-treated plates and an untreated COP plate with respect to hMSC growth kinetics and metabolic profile. The cell counting data, indirect proliferation and the lactate production levels suggest that hMSC growth kinetics appear to be favoured when seeded in COP plates coated with TA (cold plasma deposition), TB (collagen) and TC (fibronectin) using a novel plasma process. The evidence seems to be associated with the successful deposition of biomaterials used in this study, namely collagen and fibronectin, responsible for promoting cell adhesion and proliferation. These results demonstrate the applicability of plasma coated COP coated plates for cell culture and other potential uses. The concept of sensitive therapeutics being exposed to fewer materials found within the manufacturing supply chain may help reduce unwanted variances in manufacturing, however, a full line of COP-based single use systems is not available to test. Likewise, most sensitive biologics are routinely exposed to polycarbonate, polystyrene, ethyl vinyl acetate, poly vinyl chloride and others during research and development phases, exerting potentially an under-recognized biological effect from particulate and/or leachable chemical components.

## Data Availability

All data is made available and presented in the manuscript.
